# Supplementary material for: Optical Windows for Transcranial Brain Imaging in Living Mice: Skull Thinning, Clearing, and Beyond
Source: Adv Sci (Weinh). 2026 Jun 25;13(52):e76237. doi: 10.1002/advs.76237 (PMC13336979; doi:10.1002/advs.76237)
Supplement: Supplementary file 1 [file ADVS-13-e76237-s001.docx]

**Figure. S1 Quantitative signal intensity analysis and image processing for thinned skull, PoRTS, OC and TC windows.** **a.** Illustration of image processing with discontinuous imaging power. **b.** Illustration of image processing with normalized excitation power changes. **c.** Illustration of image processing with normalized exponential decay. All the images are on the same gray scale. **d.** Plot of GFP fluorescence intensity versus imaging depth of each transcranial window along Figure. S1b.

**Figure. S2 Representative images of mouse under thinned skull window with dura inflammation.** **a.** Image of GFP+ cells in dura space at different days for thinned skull with and without inflammation. The number of GFP+ cells increases significantly at day 4 and day 7 in the thinned skull with inflammation group. **b.** Statistic analysis of GFP+ cell number of different transcranial windows. (5 mice in thinned skull with inflammation group, 4 mice in thinned skull without inflammation group, 4 mice in OC group, 5 mice in TC group, 3 mice in PoRTS group). Data were present as mean ± SEM. Two-way ANOVA with Tukey’s multiple comparisons test. Only statistic comparison between thinned skull with and without inflammation is shown. ns is not shown. **c.** Comparison of skull growth and signal decay for thinned skull window with and without dura inflammation. Data were present as mean $\pm$ SEM. Unpaired t-test. ns is not shown. *p<0.05; **p<0.01; ***p<0.001; ****p<0.0001.

**Figure. S3 Optical clearing effect on aged mouse skull with vessels within the spongy bone.** **a.** x-z image of aged *Cx3cr1^GFP/+^* mouse skull with vessel in the spongy bone. Abundant vessel structures (red) and immune cells (green) appears inside the middle of SHG of skull (gray). **b.** x-y image of the plane indicated by the yellow dashed line in Fig. S3aS3a. **c.** Maximum projection image of intact skull before (left) and after clearing (right). Imaging power is 40 mW (0-50um) and 80 mW (50-150um). All the images are on the same gray scale.

**Figure. S4 Effects of skull regrowth on signal level and image resolution.** **a.** Maximum projection image of 0-50um below the pia of mouse with intact clearing window at day 0 (left) and day 21 (middle). Imaging with 2 times higher power is shown on the right. **b.** Maximum projection image of 50-150um below the pia of mouse with intact clearing window at day 0 (left) and day 21 (middle). Imaging with 2 times higher power is shown on the right. **c.** Maximum projection image of 150-250um below the pia of mouse with intact clearing window at day 0 (left) and day 21 (middle). Imaging with 2 times higher power is shown on the right.

**Figure. S5 Representative images of skull regrowth and corresponding signal decreases for thinned skull and PoRTS windows.** **a-b.** Maximum projection image of thinned skull (a) and PoRTS (b) window along y-axis on a *Cx3cr1^GFP/+^* mouse from day 0 to day 21. Skull is shown by the SHG signal (gray) and microglia is shown by GFP (green). **c-d.** Plot of GFP fluorescence intensity versus imaging depth under normalized excitation power changes for thinned skull (c) and PoRTS (d) window from day 0 to day 21.

**Figure. S6 Representative images of skull regrowth and corresponding signal decreases for OC and TC windows.** **a-b.** Maximum projection image of OC (a) and TC (b) window along y-axis on a *Cx3cr1^GFP/+^* mouse from day 0 to day 21. Skull is shown by the SHG signal (gray) and microglia is shown by GFP (green). **c-d.** Plot of GFP fluorescence intensity versus imaging depth under normalized excitation power changes for OC (c) and TC (d) window from day 0 to day 21.

**Figure. S7 Effects of re-thinning on the thinned skull window at day 14.** **a.** Maximum projection image of thinned skull window at day 0, day 14 and day 14 after re-thinning along y-axis on a same *Cx3cr1^GFP/+^* mouse. Skull is shown by the SHG signal (gray) and microglia is shown by GFP (green). All images are on the same gray scale. **b.** Skull thickness at day 0, day 14 and day 14 after re-thinning. **c.** Normalized intensity of day 0, day 14 and day 14 after re-thinning.

**Figure. S8 Effects of re-clearing on the OC window.** **a**, Maximum projection image of OC window at day 0, day 21 and day 21 re-clearing along y-axis on a same *Cx3cr1^GFP/+^* mouse. Skull is shown by the SHG signal (gray) and microglia is shown by GFP (green). All the images are on the same gray scale. **b**. Skull thickness at day 0, day 21 and day 21 after re-clearing. **c**. Normalized intensity of day 0, day 21 and day 21 after re-clearing.

**Figure. S9 Effects of re-thin and re-clearing on the TC window.** **a**, Maximum projection image of TC window at day 0, day 21 and day 21 re-thinned and re-clearing along y-axis on a same *Cx3cr1^GFP/+^* mouse. Skull is shown by the SHG signal (gray) and microglia is shown by GFP (green). All the images are on the same gray scale. **b.** Skull thickness at day 0, day 21 and day 21 after re-thinning and re-clearing. **c.** Normalized intensity of day 0, day 21 and day 21 after re-thinning and re-clearing.

**Figure S10. Comparison of imaging depth between thinned skull window, PoRTS and OC window. a.** Representative images of microglia at varying imaging depths (50 µm, 100 µm, 150 µm, and 200 µm) from the same mouse under the thinned skull window and after the implementation of the PoRTS window. **b.** Representative images of microglia at varying imaging depths (50 µm, 100 µm, 150 µm, and 200 µm) from the same mouse under the OC and after the implementation of the thinned skull window.

**Figure. S11 Comparison of fluorescence intensity of different transcranial windows.** Data are normalized with the mean value of thinned skull window. (9 mice for thinned skull, 3 mice for PoRTS, 3 mice for OC, and 5 mice for TC). Statistical analysis was performed using one-way ANOVA with Dunnett’s multiple comparisons test.

**Figure. S12 Depth-dependent aberration measurements from wavefront sensor–based AO‑TPEFM through the thinned‑skull window.** a-c. Images of the guide star (red arrowhead), corresponding wavefront sensor (WFS) readouts, reconstructed wavefronts, and Zernike coefficients at depths of 50 µm (a), 150 µm (b), and 250 µm (c) below the pia. The unit of the wavefront color bar is rad. In the Zernike coefficient plots (right), the x-axis represents the Zernike term number and the y-axis represents the normalized coefficient value.

**Figure. S13 Depth-dependent aberration measurements from wavefront sensor–based AO‑TPEFM through the PoRTS window.** a-c. Images of the guide star (red arrowhead), corresponding wavefront sensor (WFS) readouts, reconstructed wavefronts, and Zernike coefficients at depths of 50 µm (a), 150 µm (b), and 250 µm (c) below the pia. The unit of the wavefront color bar is rad. In the Zernike coefficient plots (right), the x-axis represents the Zernike term number and the y-axis represents the normalized coefficient value.

**Figure. S14 Depth-dependent aberration measurements from wavefront sensor–based AO‑TPEFM through the OC window.** a-c. Images of the guide star (red arrowhead), corresponding wavefront sensor (WFS) readouts, reconstructed wavefronts, and Zernike coefficients at depths of 50 µm (a), 150 µm (b), and 250 µm (c) below the pia. The unit of the wavefront color bar is rad. In the Zernike coefficient plots (right), the x-axis represents the Zernike term number and the y-axis represents the normalized coefficient value.

**Figure. S15 Depth-dependent aberration measurements from wavefront sensor–based AO‑TPEFM through the TC window.** a-c. Images of the guide star (red arrowhead), corresponding wavefront sensor (WFS) readouts, reconstructed wavefronts, and Zernike coefficients at depths of 50 µm (a), 150 µm (b), and 250 µm (c) below the pia. The unit of the wavefront color bar is rad. In the Zernike coefficient plots (right), the x-axis represents the Zernike term number and the y-axis represents the normalized coefficient value.

**Figure. S16 Comparison of maximum intensity and integral intensity value of simulated PSF from the measured wavefront with WFS based AO-TPEFM.** Intensity values are normalized with PSF intensity value from a perfect plane wavefront

**Figure. S17 High-resolution deep brain imaging with ALPHA-FSS through the thinned skull window.** a. Two-photon image of thinned skull window along y-axis on a *Cx3cr1^GFP/+^* mouse under system aberration correction (left) and full AO correction (right) by two-photon ALPHA-FSS system. Same skull images are shared for no AO and AO correction for representation. All the images are on the same gray scale. b. AO correction phase pattern calculated from PSF at different depth indicated by the red dashed line in (a). c. MIP images of microglia used as guiding star at corresponding depth shown without (left) and with (right) AO correction. All the images are on the same gray scale.

**Figure. S18 High-resolution deep brain imaging with ALPHA-FSS through the PoRTS window.** a. Two-photon image of thinned skull window along y-axis on a *Cx3cr1^GFP/+^* mouse under system aberration correction (left) and full AO correction (right) by two-photon ALPHA-FSS system. Same skull images are shared for no AO and AO correction for representation. All the images are on the same gray scale. b. AO correction phase pattern calculated from PSF at different depth indicated by the red dashed line in (a). c. MIP images of microglia used as guiding star at corresponding depth shown without (left) and with (right) AO correction. All the images are on the same gray scale.

**Figure. S19 High-resolution deep brain imaging with ALPHA-FSS through the OC window.** a. Two-photon image of thinned skull window along y-axis on a *Cx3cr1^GFP/+^* mouse under system aberration correction (left) and full AO correction (right) by two-photon ALPHA-FSS system. Same skull images are shared for no AO and AO correction for representation. All the images are on the same gray scale. b. AO correction phase pattern calculated from PSF at different depth indicated by the red dashed line in (a). c. MIP images of microglia used as guiding star at corresponding depth shown without (left) and with (right) AO correction. All the images are on the same gray scale.

**Figure. S20 Representative images showing inhibition of skull regrowth by locally applied dexamethasone ointment.** **a.** Maximum projection image of thinned skull window applied with dexamethasone ointment along y-axis on a *Cx3cr1^GFP/+^* mouse from day 0 to day 28. Skull is shown by the SHG signal (gray) and microglia is shown by GFP (green). **b.** Plot of GFP fluorescence intensity versus imaging depth under normalized excitation power changes from day 0 to day 28.

**Figure. S21 Representative images showing inhibition of skull regrowth by locally applied hydrocortisone ointment.** **a.** Maximum projection image of thinned skull window applied with hydrocortisone ointment along y-axis on a *Cx3cr1^GFP/+^* mouse from day 0 to day 28. Skull is shown by the SHG signal (gray) and microglia is shown by GFP (green). **b.** Plot of GFP fluorescence intensity versus imaging depth under normalized excitation power changes from day 0 to day 28.

**Figure. S22 Representative images showing activated microglia following local GC application.** **a.** Loss of higher order processes of microglia after 7 days of delivery of dexamethasone/hydrocortisone. **b.** Relative ramification index changes of same microglia before and after 7 days for control group and GC delivery group. **c.** Relative surveillance area changes of same microglia before and after 7 days for control group and GC delivery group. Data were present as mean $\pm$ SD. RM Two-way ANOVA multiple comparisons test. ****p<0.0001. ns is not shown. **d.** Contingency table of ratio between stationary and dynamic microglia for control group and GC delivery group (3 mice in the control group, 2 mice in GC group). Fisher’s exact test is used. *p<0.05.

**Figure. S23 Schematic illustration of the procedure for applying a dexamethasone‑loaded sponge to the thinned‑skull window and performing repeated *in vivo* brain imaging.**

**Figure. S24 Representative images showing inhibition of skull regrowth by a locally applied sponge loaded with 0.0135% dexamethasone solution.** **a.** Maximum projection image of thinned skull window applied with dexamethasone solution along y-axis on a *Cx3cr1^GFP/+^* mouse from day 0 to day 28. Skull is shown by the SHG signal (gray) and microglia is shown by GFP (green). **b.** Plot of GFP fluorescence intensity versus imaging depth under normalized excitation power changes from day 0 to day 28.

**Figure. S25 Schematic illustration of the procedure for applying a dexamethasone‑loaded sponge to the thinned‑skull window and performing repeated *in vivo* brain imaging.**

**Figure. S26 Representative images showing inhibition of skull regrowth by locally applied hydrogel loaded with dexamethasone solution.** **a.** Maximum projection image of thinned skull window applied with hydrogel along y-axis on a *Cx3cr1^GFP/+^* mouse from day 0 to day 35. Skull is shown by the SHG signal (gray) and microglia is shown by GFP (green). **b.** Plot of GFP fluorescence intensity versus imaging depth under normalized excitation power changes from day 0 to day 35.

**Figure. S27 Hydrogel-dexamethasone (0.0135%) delivery induces no detectable effect on microglial morphology or dynamics. a.** Relative surveillance area changes of same microglia before and after 7 days for control group and hydrogel-dexamethasone delivery group. One-way ANOVA multiple comparisons test with Dunn’s multiple comparisons test. **b.** Relative ramification index changes of same microglia before and after 7 days for control group and hydrogel-dexamethasone delivery group. One-way ANOVA multiple comparisons test with Dunn’s multiple comparisons test. **c.** Comparison of ratio of stationary and dynamic microglia changes between control group and hydrogel-dexamethasone group at different depths. Data were present as mean $\pm$ SD. Fisher’s exact test is used. Only the first ns is shown.

**Table S1 Comparison between transcranial windows**

a, Imaging intensities are normalized to thinned skull window.

b, Aberration (RMS) value measured at 50, 150, 250 μm below dura.
